# *Plasmodium vivax* malaria: An unusual presentation

**DOI:** 10.4103/0972-5229.56059

**Published:** 2009

**Authors:** Prasad Kasliwal, Manimala S. Rao, Rash Kujur

**Affiliations:** **From:** Department of Critical Care, Yashoda Hospital, Somajiguda, Hyderabad, India

**Keywords:** Acute respiratory distress syndrome, *Plasmodium vivax*, PfHRP-2, NIV

## Abstract

Acute renal failure, disseminated intravascular coagulation (DIC), acute respiratory distress syndrome (ARDS), hypoglycemia, coma, or epileptic seizures are manifestations of severe Plasmodium falciparum malaria. On the other hand, Plasmodium vivax malaria seldom results in pulmonary damage, and pulmonary complications are exceedingly rare. We report the case of a 42-year-old male living in a malaria-endemic area who presented with ARDS and was diagnosed as having Plasmodium vivax malaria. A diagnosis of Plasmodium vivax malaria was established by a positive Plasmodium LDH immunochromatographic assay while a negative PfHRP2 based assay ruled out P. falciparum malaria. After specific anti-plasmodial therapy and intensive supportive care, the patient recovered and was discharged from hospital. The use of NIPPV in vivax-malaria related ARDS was associated with a good outcome.

## Introduction

Malaria is a devastating parasitic disease transmitted through the bite of an infected Anopheles mosquitoes. *Plasmodium vivax* is the most common of the four human malaria species. Symptoms of *Plasmodium vivax* malaria are similar to those of other types of malaria and include cyclical fever with chills, headache, weakness, vomiting, and diarrhoea. Other more severe manifestations like acute renal failure, DIC, ARDS, hypoglycemia, coma, or epileptic seizures are seen with *Plasmodium falciparum* malaria and are seldom associated with *Plasmodium vivax*. Non cardiogenic pulmonary edema, manifested as ARDS, is observed in 4–18% of the cases of uncomplicated *Plasmodium falciparum* malaria with a high rate of mortality while it is uncommonly seen with *Plasmodium vivax*. We report a case of ARDS complicating infection with *Plasmodium vivax* malaria.

## Case Report

A 42-year-old male patient was admitted with fever, chills, dry cough, headache, and body pain for 1 week. He was a known hypertensive taking losartan 50 mg daily.

On admission, he was conscious, oriented, and a general examination revealed a fever (99.6°F), mild hypertension (140/90 mm Hg), a pulse rate of 88 beats/min, and RR of 22/min with oxygen saturation of 99% on room air. Systemic examination was normal. Laboratory investigations were normal except for mild thrombocytopenia (platelet count = 130 × 109/L). The peripheral blood smear showed trophozoites and schizonts of *Plasmodium vivax*. A Plasmodium LDH immunochromatographic assay was positive for *Plasmodium vivax*. Specific anti-plasmodial therapy was started with an injection of Artesunate 120 mg IV loading dose followed by 60 mg IV daily along with supportive therapy.

**Figure 1:**
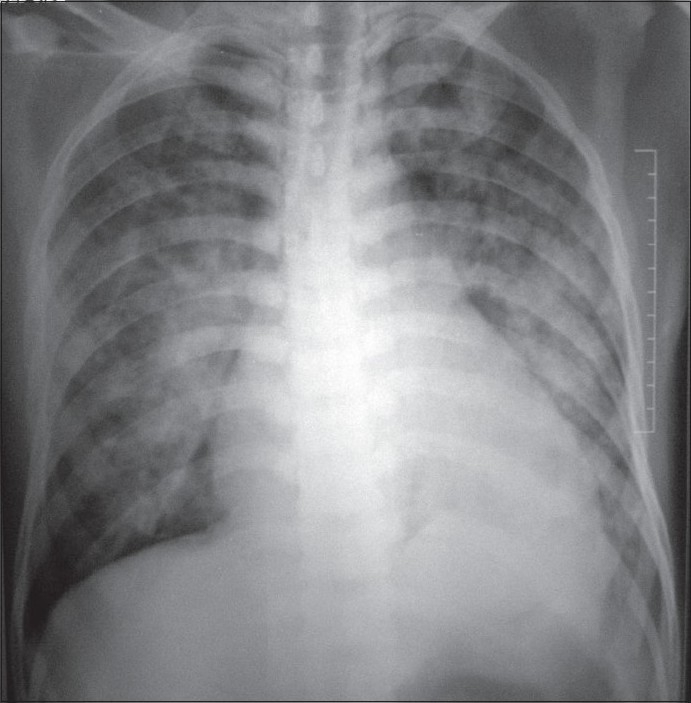
ARDS in vivax malaria

On the third day of admission, the patient developed dyspnea and tachypnea with a respiratory rate of 45 to 50 /min and pulse oximetry showed decreased oxygen saturation (86% breathing on room air). He was shifted to the Intensive Care Unit. The chest X-ray showed bilateral non homogenous opacities consistent with ARDS [Figure 1] and arterial blood gas (ABG) analysis showed pH-7.42, pO_2_-64.7 mmHg, pCO_2_-33.2 mmHg, HCO_3_-21.8 mmol/L, and a PaO_2_/FiO_2_ ratio of 161.7. A 2D Echo revealed normal cardiac function. A negative PfHRP2 assay ruled out *P. falciparum* infection. The patient's respiration was supported by non invasive positive pressure ventilation (NIPPV) with an IPAP of 12 cm H_2_O, an EPAP of 8 cmH_2_ O, and oxygen flow of 6 L/min to reduce the work of breathing. In view of a low PaO_2_/FiO_2_ ratio (<200), an injection of quinine 600 mg IV every 8 hours was added to the anti-plasmodium therapy. Central venous pressure was normal (8 cm H_2_O) and the patient was hemodynamically stable. Blood and urine cultures were sterile. An ABG done after 2 hours of NIPPV showed pH-7.38, pO_2_-120 mmHg, pCO_2_-38.4 mmHg, and HCO_3_-22.2 mmols/L.PaO_2_/FiO_2_ ratio increased to 240.

On Day 5 of intensive care, a significant improvement was seen on the chest X-ray while assisted ventilation was removed on Day 7. The patient was afebrile. Oxygen saturation was maintained using a face mask with an oxygen flow of 5 lit/min. The patient was discharged from the hospital after 14 days and Tab. Primaquine was given for 14 days for prevention of a relapse. On the follow-up visit after 15 days, the patient was asymptomatic with minimal residual findings seen on a chest X-ray.

## Discussion

This case represents one of the rare occasions where respiratory complication was observed with *Plasmodium vivax* malaria. The presence of trophozoites and schizonts of *Plasmodium vivax* on the peripheral smear and negative PfHRP2 assay along with the absence of cardiac factors for pulmonary edema establishes the diagnosis of ARDS due to *Plasmodium vivax* malaria. A PfHRP2 immunochromatic assay is proven to be a reliable method for diagnosis of *Plasmodium falciparum* malaria with a sensitivity of approx 0.001 % parasitaemia.[1]

There are only a few well-documented reports of ARDS in *Plasmodium vivax* infected cases. Tanios, *et al.* had seen a case of *Plasmodium vivax* complicated by ARDS in 2001. Kochar reported 11 cases of severe vivax malaria in 2005.[2,3]

The mechanisms underlying lung damage caused by Plasmodia are not well understood. Red blood cells parasitized by *Plasmodium vivax* do not cytoadhere to endothelial cells; thus, the occurrence of ARDS in benign malaria suggests that lung injury in malaria cases is also determined by causes other than microvascular sequestration of parasitized red blood cells. Anstey, *et al.* suggest that lung monocyte accumulation occurs in vivax and ovale malaria, as well as in falciparum malaria, with intravascular inflammatory changes contributing to impaired gas transfer and respiratory manifestations.[4] In fact, along with alveolar epithelial inflammation, ARDS has been associated with more systemic inflammatory response systems including activation of neutrophils and cytokines. The frequent onset of ARDS after starting antimalarial treatment may reflect a post-treatment exacerbation of inflammatory response mediated by proinflammatory cytokine release. Thus, the parasite probably triggers a hyperimmune response with resultant lung injury.

Recent *in vitro* but not *in vivo* data suggest that *Plasmodium vivax*-infected red cells may cytoadhere to the endothelial cell ligand chondroitin sulfate A (CSA).[5,6] Apart from the placenta, the other human endothelial cells known to express CSA are the lung and brain and this may explain the occurrence of ALI/ARDS and cerebral malaria in patients with vivax malaria.

NIPPV has revolutionized the management of acute respiratory failure and it can decrease the endotracheal intubation rates and even mortality. NIPPV has been successfully used in ARDS, which are rapidly reversible with treatment.[7] In this case, the patient developed symptoms of early ARDS after institution of antimalarial drugs for which he was initiated on NIPPV and adjunctive therapy; there was consistent improvement and an endotracheal intubation (and its subsequent complications) was prevented.

To conclude, it may be suggested that vivax malaria can cause ARDS and this point should be kept in mind while treating such patients. However, it is imperative to exclude associated mixed infections (*P. falciparum* or bacterial) or treat them simultaneously. The use of NIPPV in *Plasmodium vivax* related ARDS is associated with a good outcome.
